# The spectrum of paediatric rheumatic diseases in two tertiary centres in Cape Town, South Africa

**DOI:** 10.1186/1546-0096-12-S1-P155

**Published:** 2014-09-17

**Authors:** Lawrence O Okong'o, Christiaan Scott

**Affiliations:** 1 Paediatric Rheumatology, Red Cross Children's Hospital, University Of Cape Town, Cape Town, South Africa

## Introduction

Access to paediatric rheumatology services in sub-Saharan Africa is currently very limited with major challenges such as lack of trained personnel, diagnostic and therapeutic resources. Knowledge of the spectrum and incidence of paediatric rheumatologic diseases is important to aid in the advocacy for making paediatric rheumatology more visible and to improve access to the services in areas such as sub-Saharan Africa where the greatest unmet needs exist. However, data on the occurrence and incidence of paediatric rheumatologic diseases in Africa is scant. Single centre registry studies studies may help bridge this gap and guide planning interventions to address the diagnostic, therapeutic and human resource needs.

## Objectives

To determine the spectrum and frequency of diseases seen in the paediatric rheumatology service of two tertiary health care facilities in Cape Town, South Africa.

## Methods

We reviewed patient folders and the electronic data base of the department of paediatric rheumatology at the Red Cross children’s and Groote Schuur hospitals in Cape Town. The demographic features and diagnosis for patients seen between 2010 and May 2014 was extracted, analyzed and descriptive statistics presented using StataIC 11 software.

## Results

A total of 462 patients were in the data base; 264 (57.8%) female. The median age at first presentation at our centre was 10 (IQR 6-12.8) years. One hundred and fifty four (33.3%) were diagnosed with JIA. Other notable diagnoses included HIV associated arthritis 15 (3.3%), neutrophillic dermatoses 4 and periodic fever syndromes 6 cases. Less common conditions seen included fibrodysplasia ossificans progressiva 3, aicardi guttierrez syndrome 2 and poncet’s disease 4 cases. The findings are summarized in the table below.

**Table 1:** Frequency table of category of diseases seen

Diagnosis	Frequency	Percent	Cumulative
JIA	154	33.3	33.3

Systemic CTDs	48	10.4	43.7

Uveitis	15	3.25	46.97

Vasculitis	22	4.8	51.77

Arthritis, HIV	15	3.25	55.02

Arthralgia other arthritis	81	17.5	72.52

Pain, CRPS and other	49	10.6	83.12

Other	78	16.9	100

Total	462	100	

CTD=Connective tissue disease

## Conclusion

A wide spectrum of paediatric rheumatologic diseases was seen in this study setting. JIA was the most frequent diagnosis at 33.3% of cases. Connective tissue diseases e.g SLE, JDM and scleroderma for which scant reports exist from Africa comprised a significant part of the work load (10.4%). Vasculitis 22 (4.8%) of the cases may have been underestimated as most cases of henoch schonlein and kawasaki’s disease were followed up in the general paediatrics department. Cross sectional studies such as this could aid in understanding the scope of the problem of paediatric rheumatologic diseases, and in guiding the planning and identification of resource needs as well as preparation of practice guidelines.

## Disclosure of interest

None declared.

